# Incretin-based agents in type 2 diabetic patients at cardiovascular risk: compare the effect of GLP-1 agonists and DPP-4 inhibitors on cardiovascular and pancreatic outcomes

**DOI:** 10.1186/s12933-017-0512-z

**Published:** 2017-03-01

**Authors:** Zeqing Zhang, Xi Chen, Puhan Lu, Jianhua Zhang, Yongping Xu, Wentao He, Mengni Li, Shujun Zhang, Jing Jia, Shiying Shao, Junhui Xie, Yan Yang, Xuefeng Yu

**Affiliations:** Division of Endocrinology, Department of Internal Medicine, Tongji Hospital, Tongji Medical College, Huazhong University of Science and Technology, 1095 Jiefang Avenue, Wuhan, 430030 Hubei Province People’s Republic of China

**Keywords:** Glucagon-like peptide-1 agonists, Dipeptidyl peptidase-4 inhibitors, Type 2 diabetes mellitus, Cardiovascular outcomes, Acute pancreatitis

## Abstract

**Background:**

Incretin-based agents, including dipeptidyl peptidase-4 inhibitors (DPP-4Is) and glucagon-like peptide-1 agonists (GLP-1As), work via GLP-1 receptor for hyperglycemic control directly or indirectly, but have different effect on cardiovascular (CV) outcomes. The present study is to evaluate and compare effects of incretin-based agents on CV and pancreatic outcomes in patients with type 2 diabetes mellitus (T2DM) and high CV risk.

**Methods:**

Six prospective randomized controlled trials (EXMAINE, SAVOR-TIMI53, TECOS, ELIXA, LEADER and SUSTAIN-6), which included three trials for DPP-4Is and three trials for GLP-1As, with 55,248 participants were selected to assess the effect of different categories of incretin-based agents on death, CV outcomes (CV mortality, major adverse CV events, nonfatal myocardial infarction, nonfatal stroke, heart failure hospitalization), pancreatic events (acute pancreatitis and pancreatic cancer) as well as on hypoglycemia.

**Results:**

When we evaluated the combined effect of six trials, the results suggested that incretin-based treatment had no significant effect on overall risks of CV and pancreatic outcomes compared with placebo. However, GLP-1As reduced all-cause death (RR = 0.90, 95% CI 0.82–0.98) and CV mortality (RR = 0.84, 95% CI 0.73–0.97), whereas DPP-4Is had no significant effect on CV outcomes but elevated the risk for acute pancreatitis (OR = 1.76, 95% CI 1.14–2.72) and hypoglycemia (both any and severe hypoglycemia), while GLP-1As lowered the risk of severe hypoglycemia.

**Conclusions:**

GLP-1As decreased risks of all-cause and CV mortality and severe hypoglycemia, whereas DPP-4Is had no effect on CV outcomes but increased risks in acute pancreatitis and hypoglycemia.

**Electronic supplementary material:**

The online version of this article (doi:10.1186/s12933-017-0512-z) contains supplementary material, which is available to authorized users.

## Background

The risk of cardiovascular (CV) events is two to four times higher in patients with type 2 diabetes mellitus (T2DM) compared with those without diabetes [1–4] and also a major cause of death for these patients [5], indicating that efficient glucose management may improve CV outcomes. However, some antidiabetic drugs such as thiazolidinediones had been reported to increase incidence of heart failure [6–8]. Motivated by concerns regarding the potential association between antidiabetic agents and adverse CV outcomes, the Food and Drug Administration issued a guidance that any new antidiabetic agent was required to comprehensively evaluate its CV safety [9]. Notably, corresponding CV outcome trial should include subjects at high CV risk [10], which present a patient population more vulnerable, to obtain an event rate high enough to ascertain the CV safety of the agent [11].

Incretin-based agents include both dipeptidyl peptidase-4 inhibitors (DPP-4Is) and glucagon-like peptide-1 agonists (GLP-1As). DPP-4Is prevent the degradation of endogenous glucagon-like peptide-1 (GLP-1) and GLP-1As provide supra-physiological concentrations of “GLP-1 mimetics”. Both of them exert their effect by activating GLP-1 receptor for glucose control in patients with T2DM. However, DPP-4Is and GLP-1As are two different classes of molecules and they have different effects on CV outcomes [12] as well as corresponding parameters such as body weight [13]. Studies focused on CV safety are available now and results indicate that incretin-based agents have no adverse effect on CV outcomes except saxagliptin increasing risk of heart failure hospitalization [14] and certain GLP-1As have shown CV protective effect [15, 16]. Furthermore, it has been reported that incretin-based agents such as exenatide or sitagliptin may increase the risk of pancreatitis [17]. Therefore, a comprehensive assessment of incretin-based agents in combination and separately is important.

Previous meta-analysis and systematic reviews analyzed safety of incretin-based agents for CV outcomes but most of previous meta-analyses were performed based on studies with different follow-up durations, diverse populations of T2DM and most importantly, primary endpoints of these studies did not focus on CV events [18–28]. Recently, only one meta-analysis focused on DPP-4I studies with primary endpoints for CV outcomes [29], but missing important studies assessing newer GLP-1As. To update and clarify new accumulated evidence for CV and pancreatic safety of incretin-based agents, we conducted a meta-analysis using all available published trials for CV outcomes in patients with T2DM and high risk for CV diseases, to compare incretin-based agents with placebo.

## Methods

### Data sources and searches

Eligible English-language randomized controlled trials (up to October 2016) were identified through literature search with medical subject heading terms and keywords related to “randomized controlled trials”, “DPP-4Is” (“alogliptin”, “dutogliptin”, “linagliptin”, “omarigliptin”, “saxagliptin”, “sitagliptin”, “vildagliptin”) and “GLP-1As” (“albiglutide”, “dulaglutide”, “exenatide”, “liraglutide”, “lixisenatide”, “semaglutide”, “taspoglutide”) in Medline, Embase, Cochrane library and ClinicalTrials.gov. In addition, a search of the reference lists of eligible trials and conference abstracts were also conducted to supplement eligible studies.

### Study selection

**Fig. 1:**
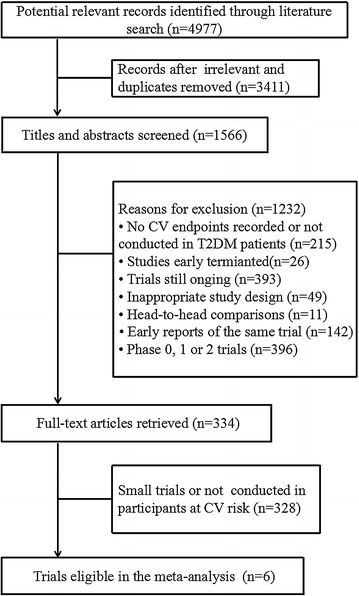
Study flow chart. *T2DM* type 2 diabetes mellitus, *CV* cardiovascular

Studies meeting the following predefined criteria were included in our analysis: [1] phase 3 and phase 4 trials; [2] compare incretin-based agents with placebo in patients with T2DM and increased risk for CV diseases; [3] follow-up for a median time of at least 52 weeks; [4] enroll at least 1000 participants; [5] report CV and other safety data for each treatment group separately. To focus on large, high quality randomized controlled trials, we excluded trials enrolling fewer than 1000 patients, or those failed to randomize properly, or not double-blinded. Head-to-head studies, early reports of the same studies and studies conducted in low CV risk patients were also excluded (Fig. 1).

Six randomized controlled trials met our selection criteria and were included in this meta-analysis: EXamination of cArdiovascular outcomes with alogliptIN versus standard of carE in patients with type 2 diabetes mellitus and acute coronary syndrome (EXAMINE) [30, 31], the Saxagliptin Assessment of Vascular Outcomes Recorded in patients with diabetes mellitus-Thrombolysis in Myocardial Infarction 53 (SAVOR-TIMI53) [14] and Trial Evaluating Cardiovascular Outcomes with Sitagliptin (TECOS) [32] tested DPP-4Is, while Lixisenatide in Patients with Type 2 Diabetes and Acute Coronary Syndrome (ELIXA) [33], Liraglutide Effect and Action in Diabetes: Evaluation of CV Outcome Results (LEADER) [15] and Semaglutide and Cardiovascular Outcomes in Patients with Type 2 Diabetes (SUSTAIN-6) [16] tested GLP-1As (Table 1).

**Table 1:** Characteristics of participants and study design of six trials

	EXAMINE	SAVOR-TIMI53	TECOS	ELIXA	LEADER	SUSTAIN-6	Overall
Study characteristics							
Number of participants	5380	16,492	14,671	6068	9340	3297	55,248
Location	898 centers in 49 countries	788 centers in 26 countries	673 centers in 38 countries	782 investigators in 49 countries	410 sites in 32 countries	230 sites in 20 countries	–
Year of publication (years)	2013/2015	2013	2015	2015	2016	2016	–
Length of follow-up (years)	1.5	2.1	3.0	2.1	3.8	2.1	2.6
Study design	Randomized, placebo-controlled						
Randomization ratio (incretin: placebo)	2701:2679	8280:8212	7332:7339	3034:3034	4688:4672	1648:1649	27,683:27,585
Interventions	Alogliptin 6.25/12.5/25 mg versus placebo	Saxagliptin 2.5/5 mg versus placebo	Sitagliptin 50/100 mg versus placebo	Lixisenatide 10–20 μg versus placebo	Liraglutide 0.6–1.8 mg versus placebo	Semaglutide 0.5/1.0 mg versus placebo	–
Baseline demographic characteristics							
Age (years)	60.9	65.0 ± 8.5	66.0 ± 8.0	60.3 ± 9.7	64.3 ± 7.2	64.6 ± 7.4	–
Male (%)	67.9	66.9	71.0	69.3	64.3	60.7	67.6
Race (%)							
White	72.7	75.2	67.9	75.2	77.5	83.0	73.9
Black	4.0	3.4	3.0	3.6	9.9	6.7	4.7
Asian	20.2	10.8	22.3	12.7	8.3	8.7	14.4
Others	3.0	10.6	6.8	8.5	4.3	2.2	7.0
BMI (kg/m^2^)	28.7 ± 11.6	31.1 ± 5.6	30.2 ± 5.7	30.2 ± 5.7	32.5 ± 6.3	32.8 ± 6.2	31.0 ± 5.7
Body weight (kg)	80.1 ± 25.9	87.9 ± 19.1	NA	84.9 ± 19.4	91.8 ± 21.0	92.1 ± 20.6	NA
Duration of diabetes (years)	7.2 ± 2.8	10.3 ± 2.8	9.4 ± 2.6	9.3 ± 8.3	12.7 ± 8.0	13.9 ± 8.1	9.7 ± 2.7
HbA_1c_ (%)	8.0 ± 1.1	8.0 ± 1.4	7.3 ± 0.7	7.7 ± 1.3	8.7 ± 1.5	8.7 ± 1.5	7.9 ± 0.3
Other medications administrated at baseline (%)							
Insulin	29.8	41.4	23.2	37.8	41.8	58.0	36.1
Metformin	66.3	69.6	81.6	63.2	76.4	73.2	73.2
Sulfonylureas	46.5	40.2	45.3	30.7	50.5	42.8	43.0
TZDs	2.4	5.9	2.7	1.4	6.1	2.3	4.0
ACEI/ARB	82.0	78.5	78.8	84.9	NA	49.8	NA
β-Blocker	82.0	61.4	63.5	84.4	55.4	57.4	65.3
Aspirin	90.7	75.2	71.0	94.4	69.8	NA	NA
Statin	90.4	78.3	79.9	92.6	72.0	72.5	80.1
Other major risk factors							
Hypertension (%)	83.1	81.4	86.0	76.4	90.0	92.8	84.4
Total cholesterol (mg/dl)	154.3 ± 43.9	NA	165.8 ± 45.3	153.0 ± 44.0	170.4 ± 45.3	NA	NA
HDL Cholesterol (mg/dl)	43.2 ± 10.6	NA	43.5 ± 12.5	43.0 ± 11.0	45.5 ± 12.3	43.7 ± 27.1	NA
LDL cholesterol (mg/dl)	78.6 ± 34.7	NA	91.0 ± 57.8	78.0 ± 35.0	89.5 ± 35.5	82.3 ± 45.6	NA
Triglycerides (mg/dl)	164.5 ± 104.0	NA	165.4 ± 99.9	164.0 ± 113.0	182.5 ± 140.0	NA	NA
Current smoker (%)	13.6	13.4	11.4	11.7	12.1	NA	NA
Corresponding changes after incretin-based agent intervention							
HbA_1c_ (%)	−0.4	−0.1	−0.3	−0.3	−0.4	−1.0 or −0.7 for different doses	−0.3
Body weight (kg)	−0.1	NA	NA	−0.7	−2.3	−4.3 or −2.9 for different doses	NA
Systolic blood pressure (mmHg)	NA	NA	NA	NA	−1.2	−2.6 or −1.3 for different doses	NA
Diastolic blood pressure (mmHg)	NA	NA	NA	NA	+0.6	NA	NA

Data are given as mean (standard deviation) or %, unless otherwise specified

*EXAMINE* EXamination of cArdiovascular outcomes with alogliptIN versus standard of carE in patients with type 2 diabetes mellitus and acute coronary syndrome, *SAVOR-TIMI53* the Saxagliptin Assessment of Vascular Outcomes Recorded in patients with diabetes mellitus-Thrombolysis in Myocardial Infarction 53, *TECOS* Trial Evaluating Cardiovascular Outcomes with Sitagliptin, ELIXA Lixisenatide in Patients with Type 2 Diabetes and Acute Coronary Syndrome, *LEADER* Liraglutide Effect and Action in Diabetes: Evaluation of CV Outcome Results, *SUSTAIN-6* Semaglutide and Cardiovascular Outcomes in Patients with Type 2 Diabetes, *BMI* body mass index, *HbA*_*1c*_ hemoglobin A_1c_, *TZD* thiazolidinedione, *ACEI/ARB* angiotensin-converting enzyme inhibitors/angiotensin receptor blocker, *NA* not applicable, *HDL* high density lipoprotein, *LDL* low density lipoprotein

### Data extraction

**Fig. 2:**
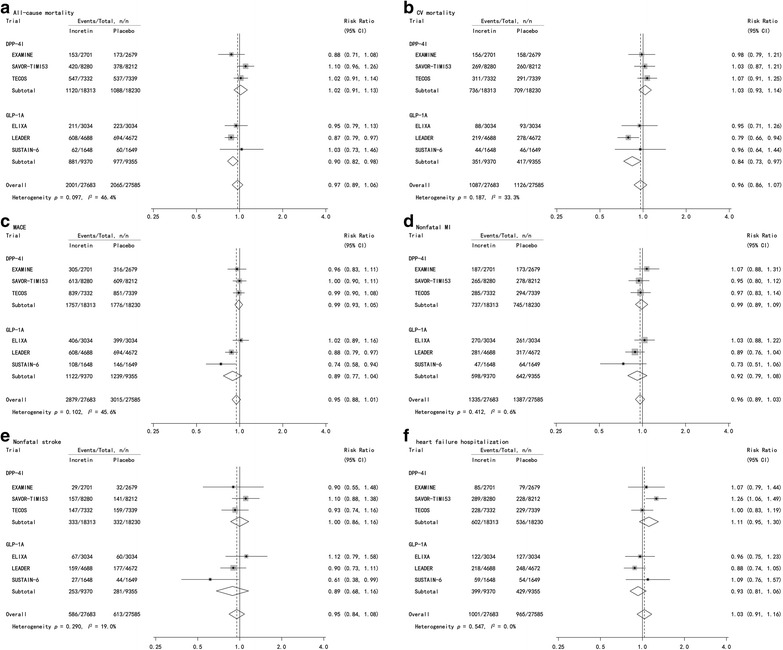
Probability of events of mortality and CV outcomes with incretin-based agents versus placebo. Effect of incretin-based agents on all-cause mortality (**a**), CV mortality (**b**), MACE (**c**), nonfatal MI (**d**), nonfatal stroke (**e**) and heart failure hospitalization (**f**) was analyzed individually. *CI* confidence interval, *DPP-4I* dipeptidyl peptidase-4 inhibitor, *GLP-1A* glucagon-like peptide-1 agonist, *CV* cardiovascular, *MACE* major cardiovascular events, *MI* myocardial infarction

**Fig. 3:**
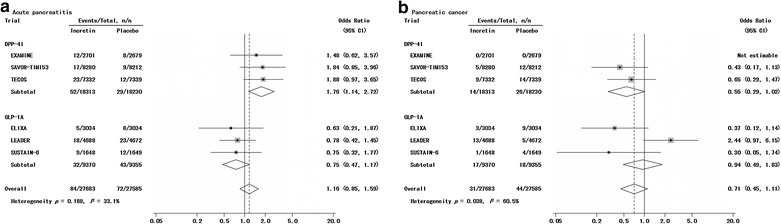
Probability of events of pancreatic outcomes with incretin-based agents versus placebo. Effect of incretin-based agents on acute pancreatitis (**a**) and pancreatic cancer (**b**) was analyzed individually. *CI* confidential interval, *DPP-4I* dipeptidyl peptidase-4 inhibitor, *GLP-1A* glucagon-like peptide-1 agonist

**Fig. 4:**
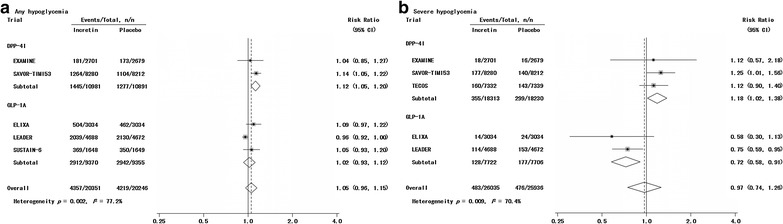
Probability of events of hypoglycemia with incretin-based agents versus placebo. Effect of incretin-based agents on any (**a**) and severe (**b**) hypoglycemia was analyzed individually. *CI* confidence interval, *DPP-4I* dipeptidyl peptidase-4 inhibitor, *GLP-1A* glucagon-like peptide-1 agonist

We abstracted the following data from each study: study characteristics (title of specific article, first author, sample size for each group, countries involved, number of study sites, year of publication, length of follow-up and study design), baseline characteristics of participants (age, sex, race, body mass index (BMI), duration of diabetes, hemoglobin A_1c_ (HbA_1c_), medications commonly administrated across groups at baseline (beyond incretin-based agents) and other major risk factors, interventions (details of incretin-based therapies, such as names of specific agents, dose) and post-intervention changes (including changes of mean concentrations of HbA_1c_, body weight, systolic pressure and diastolic pressure) (Table 1) and outcomes (absolute numbers of outcomes for both treatment arms, Figs. 2, 3, 4) were presented.

CV outcomes abstracted were as follows: all-cause mortality, CV mortality, major adverse CV events (MACE), nonfatal myocardial infarction (MI), nonfatal stroke and heart failure hospitalization. In addition, the incidences of pancreatic outcomes (acute pancreatitis and pancreatic cancer) and hypoglycemia (any hypoglycemia and severe hypoglycemia) were recorded. As definitions of these endpoints varied between studies, each endpoint was defined for each study in Additional file 1: Table S1.

Relevant data for analysis were extracted independently by two authors (ZZ and XC) using a standardized format. Discrepancies were resolved by consensus or by a senior investigator (XY).

### Data synthesis and analysis

Information for baseline characteristics were obtained with means (standard deviations) or rates from eligible studies. For most outcomes of interest, RRs (risk ratios) were pooled using both fixed- and random-effects models. Similarly, both the Cochran’s Q statistic and the *I* ^2^ measure were used to assess the heterogeneity across studies. We considered *p* value less than 0.10 in Q statistic and *I* ^2^ values of over 50% represented significant heterogeneity [34]. Therefore, evidence of heterogeneity between trials was shown for several outcomes (any hypoglycemia, severe hypoglycemia and pancreatic cancer), although findings from fixed-effects models were comparable to those from random-effects models (Additional file 1: Table S2). Additionally, the exact incretin-based therapies, lengths of follow-ups, diabetes durations of participants and achieved HbA_1c_ varied between trials. Thus, only results from random-effects models which assume these underlying impacts were present. For acute pancreatitis and pancreatic cancer, outcome data were pooled using Peto’s methods considering the relatively low event rates [35]. Subgroup analyses were performed for different types of incretin-based agents (DPP-4Is or GLP-1As) and our analyses were conducted on an intention-to-treat basis.

Sensitivity analysis was carried out when required. Alternative effect measures (RRs versus odds ratios, ORs) and pooling methods (Mantel-Hanszel versus Peto method) were tried to ensure the reliability of our results. Potential publication bias was visually evaluated with funnel plots. Furthermore, we formally assessed potential publication bias by both Begg’s [36] and Egger’s tests [37].

All tests were two-sided and *p* < 0.05 was considered significant. Analyses were done with STATA (Release 12.0; Stata Corporation, College Station, TX, USA).

## Results

As we showed in Table 1, a total of 55,248 participants were included and the average duration of follow-up (weighted by study size) was 2.6 years (median participant follow-up ranged from 1.5 years for EXAMINE to 3.8 years for LEADER). Participants were aged 60.3 to 66.0 years, with more than half were male. Moreover, most subjects included in our study were overweight or obese (BMI ranging from 28.7 to 32.8 kg/m^2^). BMI of participants were 28.7 to 31.1 kg/m^2^ in DPP-4I trials and 30.2 to 32.8 kg/m^2^ in GLP-1A trials. Similar lipid profile, blood pressure and co-administrated agents were observed among the trials. However, compared with participants in other trials, EXAMINE participants had shorter diabetes durations and lower BMI, whilst higher HbA_1c_ were recorded in participants of LEADER and SUSTAIN-6. All these trials showed lower post-intervention HbA_1c_ concentrations in participants treated with incretin-based agents than those with placebo, with mean differences ranging from −1.0 to −0.1%. The body weight were declined in all GLP-1A trials.

No overall effects of incretin-based agents on all-cause mortality (RR = 0.97, 95% CI 0.89–1.06) or CV mortality (RR = 0.96, 95% CI 0.86–1.07) were found and this was mainly due to the fact that pooling data from DPP-4I trials EXAMINE, SAVOR-TIMI53 and TECOS caused no change for the two outcomes. However, results from GLP-1A trials showed protective effects on both all-cause (RR = 0.90, 95% CI 0.82–0.98) and CV (RR = 0.84, 95% CI 0.73–0.97) mortality (Fig. 2a, b). No impacts of incretin-based agents were identified in both overall and subgroup analyses for the risk of MACE, nonfatal MI, nonfatal stroke or heart failure hospitalization (Fig. 2c–e). It is important to note that GLP-1As reduced all-cause and CV mortality were mainly caused by LEADER study, which accounted for 56.21 and 56.76% of overall results of GLP-1A trials for all-cause and CV mortality, respectively.

Incretin-based agents did not significantly affect acute pancreatitis (OR = 1.16, 95% CI 0.85–1.59, Fig. 3a) and pancreatic cancer (OR = 0.71, 95% CI 0.45–1.11, Fig. 3b). Our further analyses by comparing different classes of incretin-based agents suggested that GLP-1As were not associated with excess risks of either acute pancreatitis (OR = 0.75, 95% CI 0.47–1.17) or pancreatic cancer (OR = 0.94, 95% CI 0.49–1.83). However, DPP-4Is significantly elevated the risk of acute pancreatitis (OR = 1.76, 95% CI 1.14–2.72) and had no effect on the risk of pancreatic cancer (OR = 0.55, 95% CI 0.29–1.02).

Although pooling findings from the six trials (EXAMINE, SAVOR-TIMI53, ELIXA, LEADER and SUSTAIN-6) showed incretin-based agents were not associated with any or severe hypoglycemia (RR = 1.05, 95% CI 0.96–1.15 for any hypoglycemia, RR = 0.97, 95% CI 0.74–1.26 for severe hypoglycemia, Fig. 4), DPP-4Is increased the risk of both types of hyperglycemia and GLP-1As decreased risk for severe hypoglycemia (Fig. 4b).

In sensitivity analyses, the effect estimate was consistent when primary meta-analysis was repeated using alternative effect measures, pooling methods and statistical models. Visual inspection of funnel plots asymmetry revealed no evidence of substantial publication bias for any outcome we studied, which was further confirmed by Begg’s and Egger’s tests (Additional file 1: Table S3).

## Discussion

Incretin-based agents as an innovative class of hypoglycemic medications have several advantages over existing drugs, including glucose-dependent insulin release and weight reduction for GLP-1As or no weight gain for DPP-4Is [38]. Our present meta-analysis included data from six large CV outcome trials to compare the CV and pancreatic effects of incretin-based agents with placebo in patients with T2DM and high CV risk. The main findings of the present analysis showed that GLP-1As reduced the risks of all-cause and CV mortality and severe hypoglycemia. DPP-4Is had no adverse effect on all-cause death and CV mortality. However, they increased risks of acute pancreatitis and hypoglycemia.

The combined analysis from present data showed that incretin-based agents had no adverse effect on CV safety, which was similar to a recent study conducted in patients with T2DM and chronic kidney disease [39]. Notably, GLP-1As decreased both all-cause and CV mortality in the present study. Previous studies showed that GLP-1As had inconsistent effect on CV safety [26, 40–44]. More importantly, several studies showed that GLP-1As mainly improved certain CV risk factors such as systolic blood pressure, low density lipoprotein-cholesterol, total cholesterol, triglycerides [45], body weight and waist circumference [46], or ameliorate the CV events compared with active agent such as insulin [47] and sulphonylureas [48]. At the present time, only three high-quality CV outcome studies of GLP-1As were carried out and included in our analysis. Among them, two showed CV protective effect. The possible mechanisms underlying may be associated with reduction of body weight and blood pressure and lowering the risk for hypoglycemia. First, studies showed that all-cause mortality was significantly increased with BMI in patients with T2DM, especially when BMI ≥30 kg/m^2^ [49], and decreasing systolic blood pressure was associated with decline of all-cause death and CV mortality [50, 51]. As shown in the present studies with GLP-1As, all individuals included had average BMI above 30 kg/m^2^ and reduction of all-cause and CV mortalities was observed as decreased body weight and blood pressure were detected in all participants. For instance, body weight reduction was 2.9 or 4.3 kg and systolic pressure reduction was 1.3 or 2.6 mmHg in SUSTAIN-6 trial, 2.3 kg and 1.2 mmHg in LEADER trial and 0.7 kg in ELIXA study. Second, GLP-1As reduced severe hypoglycemia which usually associate with increasing of CV events [52, 53], and this may also account for their CV benefits.

In contrast with GLP-1As, DPP-4Is had no obvious effect on all-cause and CV mortality in patients with T2DM and high CV risk. Actually, recent data on DPP-4Is failed to show improvements with CV functions apart from T2DM patients free of a history of apparent CV diseases [54–56]. It is worthy to note that DPP-4Is elevated endogenous GLP-1 to physiological levels (10 to 25 pmol/l), whereas GLP-1As reached higher pharmacological concentrations (for example, free active liraglutide levels were in the range 60 to 90 pmol/l) and also increased endogenous GLP-1 [12, 57]. This may explain that GLP-1As have stronger effect on glycemic control and exert their CV protective effect. At the same time, the fact that DPP-4Is increased hypoglycemia may account for their non-beneficial effect on CV outcomes as GLP-1As did although endogenous GLP-1 levels were elevated.

When both classes of incretin-based treatment were combined, no significant effects on acute pancreatitis and pancreatic cancer were identified. These results were in line with a large population-based cohort study published very recently [58]. However, a refined picture was provided in our study. We found only DPP-4Is increased acute pancreatitis and there is no clear mechanism accounting for the different effects of the two types of incretin-based agents on this outcome. Notably, a recent study demonstrated that the patients with pancreatitis had reduced secretion of glucagon which might account for hypoglycemia [59]. The fact that DPP-4Is increased risk of hypoglycemia may suggest that these individuals already had certain degree of pancreas impairment, or DPP-4I molecular itself may increase risk for hypoglycemia and acute pancreatitis directly or by causing a predisposing factor for these adverse events, since DPP-4Is increased both hypoglycemia and pancreatitis as showed in our study. A head-to-head comparison of GLP-1A and DPP-4I for their effect on pancreatic safety may be helpful to clarify this issue.

The strength of our analysis is that we update the accumulated evidence to evaluate CV and pancreatic safety profile of incretin-based agents in T2DM patients at risk for CV diseases. More than 50,000 participants in six large and high-quality randomized controlled trials were included for our meta-analysis, and this ensures a strong power to detect not only their primary outcomes but small and important effects of incretin-based agents in patients.

On the other side, we must acknowledge the limitations of our meta-analysis. First, our meta-analysis were performed on summary data, thus leading to a relatively poor accuracy of assessment compared with individual-level analyses. Second, although our study showed that GLP-1As decreased all-cause death and CV mortality, the relatively short-term exposure of incretin-based agent in the studies we included may not be enough to show other potential events related to CV outcomes [38], especially for the DPP-4Is [60–68]. Third, randomized controlled trials included in our study may not reflect what happens in real world. However, results from a recent study showed the improvement in glycemia, which may affect CV and pancreatic outcomes, was the same in randomized controlled trials and the observational trial for vildagliptin [69].

## Conclusions

In conclusion, our meta-analysis reassures the CV safety of incretin-based agents among patients with T2DM and pre-existing CV risk. The impacts of these agents on CV and pancreatic outcomes seem to be class-specific, with favorable impacts on all-cause and CV mortality for GLP-1As and a detrimental one on acute pancreatitis for DPP-4Is. Moreover, hypoglycemia, as an important adverse event in the current analysis, may be related to the risk profile of CV and pancreatic outcomes. In the absence of long-term head-to-head trials, our analysis may provide insights into the comparative safety profile of DPP-4Is relative to GLP-1As. From view of this respect, GLP-1As may be prioritized over DPP-4Is in T2DM patients at CV risk.

## Additional file

**Additional file 1: Table S1.** Shows definitions of T2DM, established CV risk and other endpoints for each trial. **Table S2.** Shows overall estimates of the effects of incretin-based agents on several outcomes using fixed- and random-effects models. **Table S3.** Shows evaluation of heterogeneity and publication bias for studies included in the meta-analysis.

## Abbreviations

CV: cardiovascular
T2DM: type 2 diabetes mellitus
DPP-4I: dipeptidyl peptidase-4 inhibitor
GLP-1A: glucagon-like peptide-1 agonist
GLP-1: glucagon-like peptide-1
EXAMINE: EXamination of cArdiovascular outcomes with alogliptIN versus standard of carE in patients with type 2 diabetes mellitus and acute coronary syndrome
SAVOR-TIMI53: the Saxagliptin Assessment of Vascular Outcomes Recorded in patients with diabetes mellitus-Thrombolysis in Myocardial Infarction 53
TECOS: Trial Evaluating Cardiovascular Outcomes with Sitagliptin
ELIXA: Lixisenatide in Patients with Type 2 Diabetes and Acute Coronary Syndrome
LEADER: Liraglutide Effect and Action in Diabetes: Evaluation of CV Outcome Results
SUSTAIN-6: Semaglutide and Cardiovascular Outcomes in Patients with Type 2 Diabetes
BMI: body mass index
HbA_1c_: hemoglobin A_1c_
MACE: major adverse CV events
MI: myocardial infarction
RR: risk ratio
OR: odds ratio
CI: confidence interval

## References

[1] Huxley R, Barzi F, Woodward M (2006). Excess risk of fatal coronary heart disease associated with diabetes in men and women: meta-analysis of 37 prospective cohort studies. BMJ.

[2] Hu FB, Stampfer MJ, Haffner SM, Solomon CG, Willett WC, Manson JE (2002). Elevated risk of cardiovascular disease prior to clinical diagnosis of type 2 diabetes. Diabetes Care.

[3] Emerging Risk Factors C, Sarwar N, Gao P, Seshasai SR, Gobin R, Kaptoge S (2010). Diabetes mellitus, fasting blood glucose concentration, and risk of vascular disease: a collaborative meta-analysis of 102 prospective studies. Lancet.

[4] Meigs JB (2003). Epidemiology of cardiovascular complications in type 2 diabetes mellitus. Acta Diabetol.

[5] Matheus AS, Tannus LR, Cobas RA, Palma CC, Negrato CA, Gomes MB (2013). Impact of diabetes on cardiovascular disease: an update. Int J Hypertens.

[6] Dormandy J (2009). Safety and tolerability of pioglitazone in high-risk patients with type 2 diabetes: an overview of data from PROactive. Drug Saf.

[7] Nissen SE, Wolski K (2007). Effect of rosiglitazone on the risk of myocardial infarction and death from cardiovascular causes. N Engl J Med.

[8] Nissen SE, Wolski K (2010). Rosiglitazone revisited: an updated meta-analysis of risk for myocardial infarction and cardiovascular mortality. Arch Intern Med.

[9] Goldfine AB (2008). Assessing the cardiovascular safety of diabetes therapies. N Engl J Med.

[10] Schnell O, Standl E, Catrinoiu D, Genovese S, Lalic N, Skra J (2016). Report from the 1st cardiovascular outcome trial (CVOT) summit of the diabetes & cardiovascular disease (D&CVD) EASD study group. Cardiovasc Diabetol.

[11] Scirica BM, Bhatt DL, Braunwald E, Steg PG, Davidson J, Hirshberg B (2011). The design and rationale of the saxagliptin assessment of vascular outcomes recorded in patients with diabetes mellitus-thrombolysis in myocardial infarction (SAVOR-TIMI) 53 study. Am Heart J..

[12] Nauck M (2015). Incretin therapies—highlighting common features and differences in the modes of action of GLP-1 receptor agonists and DPP-4 inhibitors. Diabetes Obes Metab.

[13] Ahrén B, Johnson SL, Stewart M, Cirkel DT, Yang F, Perry C (2014). HARMONY 3: 104-week randomized, double-blind, placebo- and active-controlled trial assessing the efficacy and safety of albiglutide compared with placebo, sitagliptin, and glimepiride in patients with type 2 diabetes taking metformin. Diabetes Care.

[14] Scirica BM, Bhatt DL, Braunwald E, Steg PG, Davidson J, Hirshberg B (2013). Saxagliptin and cardiovascular outcomes in patients with type 2 diabetes mellitus. N Engl J Med.

[15] Marso SP, Daniels GH, Brown-Frandsen K, Kristensen P, Mann JF, Nauck MA (2016). Liraglutide and cardiovascular outcomes in type 2 diabetes. N Engl J Med.

[16] Marso SP, Bain SC, Consoli A, Eliaschewitz FG, Jodar E, Leiter LA (2016). Semaglutide and cardiovascular outcomes in patients with type 2 diabetes. N Engl J Med.

[17] Singh S, Chang HY, Richards TM, Weiner JP, Clark JM, Segal JB (2013). Glucagonlike peptide 1-based therapies and risk of hospitalization for acute pancreatitis in type 2 diabetes mellitus: a population-based matched case-control study. JAMA Intern Med.

[18] Monami M, Dicembrini I, Mannucci E (2014). Dipeptidyl peptidase-4 inhibitors and heart failure: a meta-analysis of randomized clinical trials. Nutr Metab Cardiovasc Dis.

[19] Clifton P (2014). Do dipeptidyl peptidase IV (DPP-IV) inhibitors cause heart failure?. Clin Ther.

[20] Udell JA, Cavender MA, Bhatt DL, Chatterjee S, Farkouh ME, Scirica BM (2015). Glucose-lowering drugs or strategies and cardiovascular outcomes in patients with or at risk for type 2 diabetes: a meta-analysis of randomised controlled trials. Lancet Diabetes Endocrinol.

[21] Monami M, Ahren B, Dicembrini I, Mannucci E (2013). Dipeptidyl peptidase-4 inhibitors and cardiovascular risk: a meta-analysis of randomized clinical trials. Diabetes Obes Metab.

[22] McInnes G, Evans M, Del Prato S, Stumvoll M, Schweizer A, Lukashevich V (2015). Cardiovascular and heart failure safety profile of vildagliptin: a meta-analysis of 17,000 patients. Diabetes Obes Metab.

[23] Monami M, Cremasco F, Lamanna C, Colombi C, Desideri CM, Iacomelli I (2011). Glucagon-like peptide-1 receptor agonists and cardiovascular events: a meta-analysis of randomized clinical trials. Exp Diabetes Res.

[24] Monami M, Marchionni N, Mannucci E (2009). Glucagon-like peptide-1 receptor agonists in type 2 diabetes: a meta-analysis of randomized clinical trials. Eur J Endocrinol.

[25] Kim JY, Yang S, Lee JI, Chang MJ (2016). Cardiovascular effect of incretin-based therapy in patients with type 2 diabetes mellitus: systematic review and meta-analysis. PLoS ONE.

[26] Ferdinand KC, Botros FT, Atisso CM, Sager PT (2016). Cardiovascular safety for once-weekly dulaglutide in type 2 diabetes: a pre-specified meta-analysis of prospectively adjudicated cardiovascular events. Cardiovasc Diabetol.

[27] Rehman MB, Tudrej BV, Soustre J, Buisson M, Archambault P, Pouchain D (2016). Efficacy and safety of DPP-4 inhibitors in patients with type 2 diabetes: meta-analysis of placebo-controlled randomized clinical trials. Diabetes Metab.

[28] Palmer SC, Mavridis D, Nicolucci A, Johnson DW, Tonelli M, Craig JC (2016). Comparison of clinical outcomes and adverse events associated with glucose-lowering drugs in patients with type 2 diabetes: a meta-analysis. JAMA.

[29] Abbas AS, Dehbi HM, Ray KK (2016). Cardiovascular and non-cardiovascular safety of dipeptidyl peptidase-4 inhibition: a meta-analysis of randomized controlled cardiovascular outcome trials. Diabetes Obes Metab.

[30] White WB, Cannon CP, Heller SR, Nissen SE, Bergenstal RM, Bakris GL (2013). Alogliptin after acute coronary syndrome in patients with type 2 diabetes. N Engl J Med.

[31] Zannad F, Cannon CP, Cushman WC, Bakris GL, Menon V, Perez AT (2015). Heart failure and mortality outcomes in patients with type 2 diabetes taking alogliptin versus placebo in EXAMINE: a multicentre, randomised, double-blind trial. Lancet.

[32] Green JB, Bethel MA, Armstrong PW, Buse JB, Engel SS, Garg J (2015). Effect of sitagliptin on cardiovascular outcomes in type 2 diabetes. N Engl J Med.

[33] Pfeffer MA, Claggett B, Diaz R, Dickstein K, Gerstein HC, Kober LV (2015). Lixisenatide in patients with type 2 diabetes and acute coronary syndrome. N Engl J Med.

[34] Higgins JP, Thompson SG (2002). Quantifying heterogeneity in a meta-analysis. Stat Med.

[35] Bradburn MJ, Deeks JJ, Berlin JA, Localio AR (2007). Much ado about nothing: a comparison of the performance of meta-analytical methods with rare events. Stat Med.

[36] Colin B, Begg MM (1994). Operating characteristics of a rank correlation test for publication bias. Biometrics.

[37] Guse D, Egger S, Raake A, Möller S. Web-QOE under real-world distractions: two test cases. In: Sixth international workshop on quality of multimedia experience. 2014; p. 220–5.

[38] Mannucci E, Mosenzon O, Avogaro A (2016). Analyses of results from cardiovascular safety trials with DPP-4 inhibitors: cardiovascular outcomes, predefined safety outcomes, and pooled analysis and meta-analysis. Diabetes Care.

[39] Howse PM, Chibrikova LN, Twells LK, Barrett BJ, Gamble JM (2016). Safety and efficacy of incretin-based therapies in patients with type 2 diabetes mellitus and CKD: a systematic review and meta-analysis. Am J Kidney Dis.

[40] McCormick LM, Heck PM, Ring LS, Kydd AC, Clarke SJ, Hoole SP (2015). Glucagon-like peptide-1 protects against ischemic left ventricular dysfunction during hyperglycemia in patients with coronary artery disease and type 2 diabetes mellitus. Cardiovasc Diabetol.

[41] Kumarathurai P, Anholm C, Nielsen OW, Kristiansen OP, Mølvig J, Madsbad S (2016). Effects of the glucagon-like peptide-1 receptor agonist liraglutide on systolic function in patients with coronary artery disease and type 2 diabetes: a randomized double-blind placebo-controlled crossover study. Cardiovasc Diabetol.

[42] Monami M, Dicembrini I, Nardini C, Fiordelli I, Mannucci E (2014). Effects of glucagon-like peptide-1 receptor agonists on cardiovascular risk: a meta-analysis of randomized clinical trials. Diabetes Obes Metab.

[43] Ding S, Du YP, Lin N, Su YY, Yang F, Kong LC (2016). Effect of glucagon-like peptide-1 on major cardiovascular outcomes in patients with type 2 diabetes mellitus: a meta-analysis of randomized controlled trials. Int J Cardiol.

[44] Faber R, Zander M, Pena A, Michelsen MM, Mygind ND, Prescott E (2015). Effect of the glucagon-like peptide-1 analogue liraglutide on coronary microvascular function in patients with type 2 diabetes—a randomized, single-blinded, cross-over pilot study. Cardiovasc Diabetol.

[45] Blonde L, Pencek R, MacConell L (2015). Association among weight change, glycemic control, and markers of cardiovascular risk with exenatide once weekly: a pooled analysis of patients with type 2 diabetes. Cardiovasc Diabetol.

[46] Simó R, Guerci B, Schernthaner G, Gallwitz B, Rosas-Guzmàn J, Dotta F (2015). Long-term changes in cardiovascular risk markers during administration of exenatide twice daily or glimepiride: results from the European exenatide study. Cardiovasc Diabetol.

[47] Paul SK, Klein K, Maggs D, Best JH (2015). The association of the treatment with glucagon-like peptide-1 receptor agonist exenatide or insulin with cardiovascular outcomes in patients with type 2 diabetes: a retrospective observational study. Cardiovasc Diabetol.

[48] Bain S, Druyts E, Balijepalli C, Baxter C, Currie CJ, Das R, et al. Cardiovascular events and all-cause mortality associated with sulphonylureas compared with other antihyperglycaemic drugs: a Bayesian meta-analysis of survival data. Diabetes Obes Metab. 2016 **(Epub ahead of print)**. 10.1111/dom.12821 27862902

[49] Tobias DK, Pan A, Jackson CL, O’Reilly EJ, Ding EL, Willett WC (2014). Body-mass index and mortality among adults with incident type 2 diabetes. N Engl J Med.

[50] Patel P, Ordunez P, DiPette D, Escobar MC, Hassell T, Wyss F (2016). Improved blood pressure control to reduce cardiovascular disease morbidity and mortality: the standardized hypertension treatment and prevention project. J Clin Hypertens.

[51] The SPRINT Research Group (2015). A randomized trial of intensive versus standard blood-pressure control. N Engl J Med.

[52] Zoungas S, Patel A, Chalmers J, de Galan BE, Li Q, Billot L (2010). Severe hypoglycemia and risks of vascular events and death. N Engl J Med.

[53] Bonds DE, Miller ME, Bergenstal RM, Buse JB, Byington RP, Cutler JA (2010). The association between symptomatic, severe hypoglycaemia and mortality in type 2 diabetes: retrospective epidemiological analysis of the ACCORD study. BMJ.

[54] Mita T, Katakami N, Yoshii H, Onuma T, Kaneto H, Osonoi T (2016). Alogliptin, a dipeptidyl peptidase 4 inhibitor, prevents the progression of carotid atherosclerosis in patients with type 2 diabetes: the study of preventive effects of alogliptin on diabetic atherosclerosis (SPEAD-A). Diabetes Care.

[55] Tomiyama H, Miwa T, Kan K, Matsuhisa M, Kamiya H, Nanasato M (2016). Impact of glycemic control with sitagliptin on the 2-year progression of arterial stiffness: a sub-analysis of the PROLOGUE study. Cardiovasc Diabetol.

[56] Ida S, Murata K, Betou K, Kobayashi C, Ishihara Y, Imataka K (2016). Effect of trelagliptin on vascular endothelial functions and serum adiponectin level in patients with type 2 diabetes: a preliminary single-arm prospective pilot study. Cardiovasc Diabetol.

[57] Kramer CK, Zinman B, Choi H, Connelly PW, Retnakaran R. Chronic liraglutide therapy induces an enhanced endogenous glucagon-like peptide-1 secretory response in early type 2 diabetes. Diabetes Obes Metab. 2017 **(Epub ahead of print)**. 10.1111/dom.12858 28181363

[58] Tseng CM, Liao WC, Chang CY, Lee CT, Tseng CH, Hsu YC (2016). Incretin-based pharmacotherapy and risk of adverse pancreatic events in the ethnic Chinese with diabetes mellitus: a population-based study in Taiwan. Pancreatology.

[59] Meier JJ, Giese A (2015). Diabetes associated with pancreatic diseases. Curr Opin Gastroenterol.

[60] White WB, Baker WL (2016). Cardiovascular effects of incretin-based therapies. Annu Rev Med.

[61] Ussher JR, Drucker DJ (2014). Cardiovascular actions of incretin-based therapies. Circ Res.

[62] Ayaori M, Iwakami N, UtoKondo H, Sato H, Sasaki M, Komatsu T (2013). Dipeptidyl peptidase-4 inhibitors attenuate endothelial function as evaluated by flow-mediated vasodilatation in type 2 diabetic patients. J Am Heart Assoc..

[63] Bostick B, Habibi J, Ma L, Aroor A, Rehmer N, Hayden MR (2014). Dipeptidyl peptidase inhibition prevents diastolic dysfunction and reduces myocardial fibrosis in a Mouse model of Western diet induced obesity. Metabolism.

[64] Nakagami H, Pang Z, Shimosato T, Moritani T, Kurinami H, Koriyama H (2014). The dipeptidyl peptidase-4 inhibitor teneligliptin improved endothelial dysfunction and insulin resistance in the SHR/NDmcr-cp rat model of metabolic syndrome. Hypertens Res.

[65] Zeng Y, Li C, Guan M, Zheng Z, Li J, Xu W (2014). The DPP-4 inhibitor sitagliptin attenuates the progress of atherosclerosis in apolipoprotein-E-knockout mice via AMPK- and MAPK-dependent mechanisms. Cardiovasc Diabetol.

[66] Aroor AR, Javad H, Ford DA, Nistala R, Lastra G, Manrique C (2015). Dipeptidyl peptidase-4 inhibition ameliorates western diet-induced hepatic steatosis and insulin resistance through hepatic lipid remodeling and modulation of hepatic mitochondrial function. Diabetes.

[67] Bader M, Lu HY, Huang CY, Shih CM, Chang WH, Tsai CS (2015). Dipeptidyl peptidase-4 inhibitor decreases abdominal aortic aneurysm formation through GLP-1-dependent monocytic activity in mice. PLoS ONE.

[68] Hirakawa H, Zempo H, Ogawa M, Watanabe R, Suzuki J, Akazawa H (2015). A DPP-4 inhibitor suppresses fibrosis and inflammation on experimental autoimmune myocarditis in mice. PLoS ONE.

[69] Ahrén B, Mathieu C, Bader G, Schweizer A, Foley JE (2014). Efficacy of vildagliptin versus sulfonylureas as add-on therapy to metformin: comparison of results from randomised controlled and observational studies. Diabetologia.

