# Supplementary material for: Incretin-based agents in type 2 diabetic patients at cardiovascular risk: compare the effect of GLP-1 agonists and DPP-4 inhibitors on cardiovascular and pancreatic outcomes
Source: Cardiovasc Diabetol. 2017 Mar 1;16:31. doi: 10.1186/s12933-017-0512-z (PMC5333444; doi:10.1186/s12933-017-0512-z)
Supplement: Supplementary file 1 — Additional file 1: Table S1. Shows definitions of T2DM, established CV risk and other endpoints for each trial. Table S2. Shows overall estimates of the effects of incretin-based agents on several outcomes using fixed- and random-effects models. Table S3. Shows evaluation of heterogeneity and publication bias for studies included in the meta-analysis. [file 12933_2017_512_MOESM1_ESM.doc]

**Supplementary Material:**

Supplement to Zhang Z, Chen X, Yu X et al. Incretin-based agents in type 2 diabetic patients at cardiovascular risk: compare the effect of GLP-1 agonists and DPP-4 inhibitors on cardiovascular and pancreatic outcomes.

| **Table S1**: Definitions of T2DM, established CV risk and other endpoints for each trial | | | | | | |
| --- | --- | --- | --- | --- | --- | --- |
|  | **EXAMINE** | **SAVOR-TIMI53** | **TECOS** | **ELIXA** | **LEADER** | **SUSTAIN-6** |
| T2DM | Patients with T2DM with HbA1c of 6.5 to 11.0% at screening, or if the antidiabetic regimen included insulin, HbA1c level of 7.0 to 11.0%. | Patients with T2DM with HbA_1c_ of 6.5% to 12.0%. | Patients with T2DM with HbA_1c_ of 6.5% to 8.0%. | Patients with T2DM with HbA_1c_ of 5.5% to 11.0%. | Patients with T2DM and HbA_1c_ of 7.0% or more. | Patients with T2DM and HbA1c of 7.0% or more. |
| Established CV risk | Acute coronary syndrome. | (1) At least 40 years old and have a history of a clinical event associated with atherosclerosis involving the coronary, cerebro- vascular, or peripheral vascular system; (2) at least 55 years of age (men) or 60 years of age (women) with multiple CV risk factors for vascular diseases. | At least 50 years of age and have a history of major coronary artery disease, ischemic cerebrovascular disease, or atherosclerotic peripheral arterial disease | Acute coronary syndrome. | (1) At least 50  years old and have a history of a prior CV event including cerebrovascular  disease, peripheral vascular disease, chronic renal failure, or chronic heart failure; (2) at least 60 years of age with multiple CV risk factors for vascular diseases. | (1) Age 50 years or older with documented clinical evidence of  CV disease; (2) age 60 years or older with subclinical evidence of CV diseases. |
| All-cause mortality | Deaths from any cause. | Deaths from any cause. | Deaths from any cause. | Deaths from any cause. | Deaths from any cause. | Deaths from any cause. |
| CV mortality | (1) Deaths from cardiac or cerebrovascular causes; (2) death without another known cause. | (1) Sudden cardiac death; (2) deaths caused by acute MI, heart failure, stroke, and other CV causes. | A first confirmed event of CV death, nonfatal MI, nonfatal stroke, or hospitalization for unstable angina. | Deaths from a documented CV cause (including fatal MI, pump failure, sudden death, presumed sudden death, presumed CV death, fatal stroke, fatal pulmonary embolism, procedure-related death and other CV causes). | Deaths from a documented CV cause (including (1) acute MI, cardiogenic shock, cerebrovascular event, heart failure; (2) sudden / unexpected cardiac cause, CV cause, sudden cardiac cause, other CV cause, presumed CV cause, or other CV cause). | (1) Deaths from acute MI, heart failure, stroke, CV hemorrhage, CV  procedures and other CV causes; (2) sudden cardiac death. |
| MACE | A composite of CV death, nonfatal acute MI, and nonfatal stroke. | A composite of death from CV causes, nonfatal MI, or nonfatal stroke. | A first confirmed event of CV death, nonfatal MI, nonfatal stroke, or hospitalization for unstable angina. | A first confirmed event of CV death, nonfatal MI, nonfatal stroke, or unstable angina. | A composite of CV death, nonfatal acute MI, and nonfatal stroke. | A composite of death from CV causes, nonfatal MI or nonfatal stroke. |
| Nonfatal MI | New events of nonfatal MI. | First events of nonfatal MI. | First events of nonfatal MI. | Nonfatal MI including spontaneous MI, PCI-related MI and coronary artery bypass graft–related MI. | Nonfatal MI including spontaneous MI, ST-elevation MI, non–ST-elevation MI, PCI-related MI, coronary artery bypass grafting- related MI or silent MI. | Evidence of myocardial necrosis in a clinical setting consistent with  MI. |
| Nonfatal stroke | New events of nonfatal strokes. | New events of nonfatal strokes. | New events of nonfatal strokes. | New events of nonfatal strokes. | New events of nonfatal strokes. | New events of nonfatal strokes. |
| Heart failure hospitalization | An inpatient admission or an emergency department visit of  more than 12 h with clinical manifestations of heart failure. | An event of heart failure (with clinical manifestations) which requires hospitalization or additional/increased therapy. | All episodes of suspected congestive heart failure requiring hospitalization. | Unplanned presentation to an acute care facility for an exacerbation of heart failure requiring an overnight stay which meets the following criteria: (1) symptoms of heart failure; (2) signs of heart failure; (3) treatment for heart failure. | An event with clinical manifestations of heart failure and requires additional/increased therapy. | A hospitalization of  more than 24 h with symptoms of heart failure, which requires initiation or intensification of treatment. |
| Any hypoglycemia | First events of hypoglycemia. | Any recorded blood glucose <3.0 mmol/L. | First events of hypoglycemia. | Any recorded blood glucose <3.3 mmol/L. | Hypoglycemia events including severe, documented symptomatic, asymptomatic, probable symptomatic and relative hypoglycemia. | Any recorded blood glucose < 3.1mmol/L. |
| Severe hypoglycemia | First events of severe hypoglycemia. | Defined as hypoglycemia that require assistance of another person to actively administercarbohydrate, glucagon, or other resuscitative actions. | Defined as hypoglycemia requiring the assistance of another individual. | Defined as hypoglycemia requiring assistance by another person, and/or prompt recovery after treatment. | Defined as hypoglycemia requiring the assistance of another person to administer resuscitative actions, carbohydrate, or glucagon. | First events of severe hypoglycemia. |
| Acute pancreatitis | First events of acute pancreatitis. | To confirm a diagnosis of acute pancreatitis, the following criteria must be present: (1) typical abdominal pain; (2) serum amylase and/or lipase > 3 UNL; (3) abnormal imaging consistent with acute pancreatitis. | To confirm a diagnosis of acute pancreatitis, the following criteria must be present: (1) symptoms of abdominal pain or vomiting; (2) objective evidence of pancreatic inflammation. | First events of acute pancreatitis. | First events of acute pancreatitis. | First events of acute pancreatitis. |
| Pancreatic cancer | First events of pancreatic cancers. | First events of pancreatic cancers. | All suspected or confirmed occurrences of pancreatic malignancies. | First events of pancreatic cancers. | First events of pancreatic cancers. | First events of pancreatic cancers. |
| Abbreviations: T2DM, type 2 diabetes mellitus, CV, cardiovascular; EXAMINE, EXamination of cArdiovascular outcomes with alogliptIN versusstandard of carE in patients with type 2 diabetes mellitus and acute coronary syndrome; SAVOR-TIMI53, the Saxagliptin Assessment of Vascular Outcomes Recorded in patients with diabetes mellitus–Thrombolysis in Myocardial Infarction 53; TECOS, Trial Evaluating Cardiovascular Outcomes with Sitagliptin; ELIXA, Lixisenatide in Patients with Type 2 Diabetes and Acute Coronary Syndrome; LEADER, Liraglutide Effect and Action in Diabetes: Evaluation of CV Outcome Results; SUSTAIN-6, Semaglutide and Cardiovascular Outcomes in Patients with Type 2 Diabetes; HbA_1c_, Hemoglobin A_1c_; MACE, major acute coronary events; MI, myocardial infarction; PCI, percutaneous coronary intervention; UNL, upper normal limit. | | | | | | |

| **Table S2**: Overall estimates of the effects of incretin-based agents on several outcomes using fixed- and random-effects models | | | | |
| --- | --- | --- | --- | --- |
| Outcome | Pooled RR (95% CI) | | Heterogeneity across trials | |
|  | Fixed-effect model | Random-effect model | *p* value | *I*^2^ (%) |
| All-cause mortality | 0.97 (0.91,1.02) | 0.97 (0.89,1.06) | 0.097 | 46.4% |
| CV mortality | 0.96 (0.89,1.04) | 0.96 (0.86,1.07) | 0.187 | 33.3% |
| MACE | 0.95 (0.91,1.00) | 0.95 (0.88,1.01) | 0.102 | 45.6% |
| Nonfatal MI | 0.96 (0.89,1.03) | 0.96 (0.89,1.03) | 0.412 | 0.6% |
| Nonfatal stoke | 0.95 (0.85,1.07) | 0.95 (0.84,1.08) | 0.290 | 19.0% |
| Heart failure hospitalization | 1.03 (0.95,1.13) | 1.03 (0.91,1.16) | 0.115 | 43.5% |
| Any hypoglycemia | 1.03 (0.99,1.07) | 1.05 (0.96,1.15) | 0.002 | 77.2% |
| Severe hypoglycemia | 1.01 (0.89,1.15) | 0.97 (0.74,1.26) | 0.009 | 70.4% |
| Abbreviations: CI, confidence interval; RR, risk ratio; CV, cardiovascular; MACE, major acute coronary events; MI, myocardial infarction. | | | | |

| **Table S3**: Evaluation of heterogeneity and publication bias for studies included in the meta-analysis | | |
| --- | --- | --- |
|  | *p* value of the Begg’s test | *p* value of the Egger’s test |
| All-cause mortality | 1.000 | 0.794 |
| CV mortality | 0.260 | 0.854 |
| MACE | 0.707 | 0.309 |
| Nonfatal MI | 1.000 | 0.390 |
| Nonfatal stroke | 1.000 | 0.457 |
| Heart failure hospitalization | 0.707 | 0.991 |
| Acute pancreatitis | 0.707 | 0.821 |
| Pancreatic cancer | 0.462 | 0.543 |
| Any hypoglycemia | 0.806 | 0.314 |
| Severe hypoglycemia | 0.462 | 0.585 |
| Abbreviations: CV, cardiovascular; MACE, major adverse cardiovascular events; MI, myocardial infarction. | | |
